# Generation of SARS-CoV-2 Spike Pseudotyped Virus for Viral Entry and Neutralization Assays: A 1-Week Protocol

**DOI:** 10.3389/fcvm.2020.618651

**Published:** 2021-01-15

**Authors:** Jose Manuel Condor Capcha, Guerline Lambert, Derek M. Dykxhoorn, Alessandro G. Salerno, Joshua M. Hare, Michael A. Whitt, Savita Pahwa, Dushyantha T. Jayaweera, Lina A. Shehadeh

**Affiliations:** ^1^Division of Cardiology, Department of Medicine, University of Miami Leonard M. Miller School of Medicine, Miami, FL, United States; ^2^Interdisciplinary Stem Cell Institute, University of Miami Leonard M. Miller School of Medicine, Miami, FL, United States; ^3^Dr. John T. Macdonald Foundation Department of Human Genetics, John P. Hussman Institute for Human Genomics, University of Miami Leonard M. Miller School of Medicine, Miami, FL, United States; ^4^Department of Microbiology, Immunology and Biochemistry, University of Tennessee Health Science Center, Memphis, TN, United States; ^5^Department of Microbiology and Immunology, University of Miami Leonard M. Miller School of Medicine, Miami, FL, United States; ^6^Division of Infectious Disease, Department of Medicine, University of Miami Leonard M. Miller School of Medicine, Miami, FL, United States; ^7^Peggy and Harold Katz Family Drug Discovery Center, University of Miami Leonard M. Miller School of Medicine, Miami, FL, United States

**Keywords:** SARS-CoV-2, pseudovirus, lentivirus, ACE2, TMPRSS2, furin, poloxamer 407, prostaglandin E2

## Abstract

The COVID-19 pandemic caused by the SARS-CoV-2 coronavirus requires reliable assays for studying viral entry mechanisms which remains poorly understood. This knowledge is important for the development of therapeutic approaches to control SARS-CoV-2 infection by permitting the screening for neutralizing antibodies and other agents that can block infection. This is particularly important for patients who are at high risk for severe outcomes related to COVID-19. The production of pseudotyped viral particles may seem like a daunting task for a non-virology laboratory without experience in the two most commonly used pseudotyping systems, namely retro/lentiviruses and vesicular stomatitis virus (VSV) which lacks the VSV envelope glycoprotein (VSVΔG). By incorporating the most up-to-date knowledge, we have developed a detailed, easy-to-follow novel protocol for producing SARS-CoV-2 spike-bearing pseudovirus using the VSV-ΔG system. We describe the infection assay which uses GFP fluorescence as a measure of infection in a 24-well live imaging system. We present results of our optimization of the system to enhance viral infection levels through the over-expression of human ACE2 receptor and the overexpression of at least one of two proteases - TMPRSS2 or Furin, as well as, supplementation with Poloxamer 407 (P407) and Prostaglandin E2 (PGE2) as adjuvants. We show that the system works efficiently in three unrelated, clinically relevant cell lines: human 293T (renal epithelial) cells, human Calu-3 (lung epithelial) cells, and the non-human primate (African Green Monkey) cell line, Vero-E6 (renal epithelial) cells. In addition, we have used this system to show infection of human induced pluripotent stem cell-derived cardiomyocytes (iPS-CMs). This system is efficient (virus generation, titration, and infection assays can be performed in 1 week), quantitative, inexpensive, and readily scalable for application in drug development and therapeutic screening approaches.

## Introduction

The coronavirus disease (COVID-2019) pandemic is caused by severe acute respiratory syndrome coronavirus 2 (SARS-CoV-2) (1). SARS-CoV-2 is an enveloped, positive-sense single stranded RNA virus belonging to the Betacoronavirus genus of the Coronaviridae family of viruses (2). Understanding the viral entry process is key for the development of therapeutic strategies to prevent viral spread. The spike (S) glycoprotein is present on the surface of SARS-CoV-2 and is responsible for the viral tropism through host cell recognition by binding to the human angiotensin-converting enzyme 2 (hACE2) receptor (3, 4). Coronavirus S glycoprotein is a class I viral fusion protein (5) containing a homotrimeric conformation with two functional subunits termed S1 and S2 present in the N-terminal domain (NTD) and C-terminal domain (CTD), respectively (6). The S protein interacts with hACE2 through the receptor binding domain (RBD) located in the NTD (S1) (7). Once bound to hACE2, the S1 domain is cleaved and dissociated from the protein exposing the S2 domain containing the S transmembrane domain, cytoplasmic tail, and the fusion peptide (8). Spike protein cleavage between the S1 and S2 domain is mediated by several host proteases, such as Furin or the transmembrane protease serine protease-2 (TMPRSS2). This S protein cleavage triggers conformational rearrangements of the S2 domain to activate the protein for viral membrane fusion and release of the viral genetic material into the cells (8, 9).

Recovered COVID-19 patients develop neutralizing antibodies (nAbs) against the Spike glycoprotein proving its high antigenicity (9, 10). *In vitro* cellular assays using the Spike glycoprotein are therefore used to study viral infection or Spike/hACE2 interaction. However, the Spike protein alone fails to mimic the virus-host cell interaction. On the other hand, experiments with actual pathogenic SARS-CoV-2 demands strict biosafety level-3 (BSL-3) laboratory conditions (11). The use of replication-restricted pseudoviruses bearing viral coat proteins represents a safe and useful method that has been widely adopted by virologists to study viral entry, detection of nAbs in serum samples, and therapeutic development under less stringent biosafety conditions [e.g., biosafety level-2 (BSL-2)]. Therefore, pseudotyping viral systems have been widely employed to study highly infectious and pathogenic viruses such as Ebola virus, Middle Eastern Respiratory Syndrome (MERS) virus, or SARS viruses (12–14).

Vesicular stomatitis virus (VSV) is an enveloped negative-stranded RNA virus that infects a wide range of animals and less frequently humans causing mild flu-like symptoms (15–17). The simple structure, and ability to grow in a wide range of mammalian cell types with high titer has made VSV a promising viral vector and a valuable molecular biological and virological tool (18, 19). The VSV genome encodes five main proteins: matrix protein (M), nucleoprotein (N), large polymerase protein (L), phosphoprotein (P) and glycoprotein (G) (20). The G protein interacts with host low density lipoprotein receptor (LDLR) for viral entry and cell fusion (21).

To study the entry of viruses that require BSL-3 or−4 containment, recombinant VSVs (rVSVs) in which the glycoprotein gene has been deleted and replaced by a reporter gene - Green Fluorescent Protein (GFP), Red fluorescent protein (RFP/DsRed), Secreted Embryonic Alkaline Phosphatase (SEAP), or firefly Luciferase (fLuc) as summarized in Table 1 - has provided a powerful tool that can be used in laboratories that do not have access to BSL-3 or−4 facilities (22). The advantages of the fluorescent protein reporters are that exact titers of the pseudotyped virus can be determined by counting individual cells infected by the pseudotypes. Because of the robust expression of viral proteins (including the reporter), the sensitivity is extremely high (at the single cell level). No substrates are needed and the equipment needed for analysis is a fluorescence microscope (with option of advanced live imaging system like one described here) or a flow cytometer. Both fLuc and SEAP provide a wide dynamic range when analyzing inhibitors of infection (either antiviral or neutralizing antibodies). The disadvantage is that both require additional steps to assay the reporters. In the case of the SEAP reporter, the pseudotyped virus needs to be purified by centrifugation through a sucrose cushion to pellet the virus away from the SEAP that is produced and in the culture supernatant of the pseudotyped virus. However, the cost of the SEAP reagents are significantly less than the reagents needed to assay fLuc, which is not secreted. Both of these reporters also require access to a plate reader that can analyze either luminescence (for fLuc) or absorbance (for SEAP).

**Table 1:** Reporters used with recombinant vesicular stomatitis virus (VSV) lacking the envelope glycoprotein (rVSVΔG).

**Gene reporters for pseudotyping**	**Methodology of detection**	**Advantages**	**Disadvantages**
Pseudotyped ΔG-**GFP** (G^*^ΔG-GFP) rVSV	Fluorescence	- Live-cell imaging - Cytotoxicity evaluation - Cells usable for RNA/protein work - Flourescence microscope	
Pseudotyped ΔG-**DsRed** (G^*^ΔG-DsRed) rVSV	Fluorescence	- Live-cell imaging - Cytotoxicity evaluation - Cells usable for RNA/protein work - Flourescence microscope	
Pseudotyped ΔG-**luciferase** (G^*^ΔG-luciferase) rVSV	Luminescence assay for alkaline phosphatase (AP) activity	- Low cost for kits and reagents - Plate reader	- Background for pseudoviral infection
Pseudotyped ΔG-**SEAP** (G^*^ΔG-SEAP) rVSV	Luminescence/ Colorimetric assay for alkaline phosphatase (AP) activity	- Low cost for kits and reagents - Plate reader	- Background for pseudoviral infection - Internal alkaline phosphatase (AP) activity

rVSVΔG*Reporter can be packaged and released from cells transfected with mammalian expression plasmid encoding G protein or the envelope protein from other viruses (3, 22). Thus, rVSVΔG*Reporter system can be used to produce single-round replication-restricted VSV pseudoviruses bearing any viral surface glycoprotein (especially from those that would otherwise require work under BSL-3 and BSL-4 containments) in BSL-2 laboratories (22).

In this study, we present an effective methodology for studying the role of the SARS-CoV-2 spike protein in determining cellular tropism and viral entry with potential applications for the screening of anti-SARS-CoV-2 neutralizing antibodies and alternative SARS-CoV-2 entry inhibitors. This approach is based on the combination of previously developed resources and approaches to address this important area of research in SARS-CoV-2 infection and therapeutic development. We have utilized a commercially available plasmid for the expression of a truncated GFP-bearing SARS-CoV-2 spike protein and recombinant VSVΔG particles to develop a detailed, easy-to-follow protocol for making the SARS-CoV-2 spike-bearing pseudovirus. The truncated spike protein was used because it deletes the Endoplasmic Reticulum (ER)-retention signal found in the cytoplasmic domain of the spike protein. This results in a slightly higher levels of the SARS-CoV-2 spike/VSV pseudotypes (18, 23). We describe the infection assay using GFP fluorescence as a measure of infection utilizing a live imaging system. We show that for optimal SARS-CoV-2 spike protein-based pseudovirus entry, we require the over-expression of human ACE2 receptor, and at least one of two proteases—TMPRSS2 or Furin. We show that the system works efficiently in 3 cell lines: 293T (human renal epithelial) cells, Calu-3 (human lung epithelial) cells, and the non-human primate cell line, Vero-E6 (African Green Monkey renal epithelial) cells, as well as, in human iPS-derived cardiomyocytes—all reported to be infected by SARS-CoV-2 (3, 24–27). The system is efficient, quantitative, inexpensive, and the virus generation, titration, and infection assay can be performed in 1 week making this approach amenable for use in neutralizing antibody analysis or high content therapeutic screening.

## Materials

### Cell Culture

- Embryonic kidney 293T cells (ATCC Catalog # CRL-321)
- Calu-3 (ATCC Catalog # HTB-55)
- Vero-E6 (ATCC Catalog # CRL-1586)
- hiPS-CMs were generated as previously described by our group (28)
- Dulbecco's Modified Eagle's Medium (DMEM) (ThermoFisher Catalog #11965-92)
- Minimum Essential Medium Eagle (MEM) (Sigma Aldrich Catalog #M0325)
- Fetal bovine serum (FBS) (Atlas Biologicals Catalog # FP-0500-A)
- Penicillin-Streptomycin-Glutamine (P/S/G) (100X) (ThermoFisher Catalog # 10378016)
- Trypsin-EDTA (0.25%), phenol red (ThermoFisher Catalog #25200056)
- Lipofectamine™ 2000 Transfection Reagent (ThermoFisher Catalog #11668019)
- Opti-MEM™ Reduced Serum Medium (ThermoFisher cat. #11058021)
- Sodium pyruvate solution (Sigma Aldrich Catalog #S8636)
- Sterile, 15 or 50 ml polystyrene centrifuge tubes.

### Virus, Plasmids, and Antibodies

- CAUTION! All the experiments involving viruses and human subjects must comply with national and institutional regulations for research purposes. Plasma samples from COVID-19 surviving subjects were collected under approved Institutional Review Board (IRB) protocol# 20200303
- Pseudotyped rVSVΔG-GFP*G (Kerafast catalog # EH1019)
- pCAGGS-G-Kan plasmid (Kerafast catalog # EH1017)
- pCMV14-3X-Flag-SARS-CoV-2 S plasmid (Addgene catalog # 145780) (6)
- psPAX2 GagPol plasmid (Addgene catalog # 12260)
- pMD2.GVSV-G plasmid (Addgene catalog # 12259)
- pLenti-ACE2 (Genecopoeia catalog # EX-U1285-Lv130)
- pLenti-TMPRSS2 (Genecopoeia catalog # EX-Z7591-Lv204)
- pLenti-Furin (Genecopoeia catalog # EX-F0229-Lv120)
- Anti-VSV-G antibody, clone 8G5F11 (Millipore catalog #MABF2337)
- Anti-VSV-M antibody, clone 23H12(Kerafast catalog # EB0011)
- Anti-ACE2 antibody (R&D Systems catalog #AF933)
- Anti-ACE2 antibody (R&D Systems catalog#MAB933)
- Anti-TMPRSS2 antibody (DSHB catalog#P5H9-A3)
- Anti-Furin antibody (Santa Cruz catalog#133142)
- Anti-Flag antibody (Sigma Aldrich catalog #F1804)
- Anti-c-Myc antibody (Santa Cruz catalog#sc-40).

### Chemicals

- Poloxamer 407 (Sigma Aldrich catalog #16758)
- Prostaglandin E_2_, PGE2, (Cayman chemical catalog # 14750)
- Hydroxychloroquine (Cayman chemical catalog # 14750)
- Camostat mesylate (Cayman chemical catalog # 16018)
- Pierce® RIPA Lysis and Extraction Buffer (Thermo Scientific catalog #89900)
- Sucrose (Sigma Aldrich catalog #S0389).

### qPCR Reagents

- Mirvana Paris Kit RNA and Native Protein Purification Kit (ThermoFisher—catalog #AM1556)
- High Capacity cDNA Reverse Transcription Kit (ThermoFisher—catalog #4368813)
- SsoAdvanced Universal Probes Supermix (Bio-Rad—catalog # 1725284)
- GAPDH Taqman assay (Thermofisher—assay ID # Hs02786624_g1)
- GFP Taqman assay (Thermofisher—assay ID # Mr03989638_mr).

### Imaging System

- IncuCyte® ZOOM live-cell analysis system (Sartorius)
- Fluorescence Microscope: ZEISS Axio Observer (Zeiss).

### Equipment and Cautions

- Personal Protective Equipment. For all the procedures with pseudovirus and lentivirus generation use laboratory coats, latex gloves, face mask, and protective eye wear
- CAUTION! Use a solution of Sodium hypochlorite 10% to wash or discard all materials that are in contact with pseudovirus and lentivirus.

## Procedures

### Production of VSVΔG Bearing SARS-CoV-2 Spike-Δ19 (Truncated Spike)

**Figure 1:**
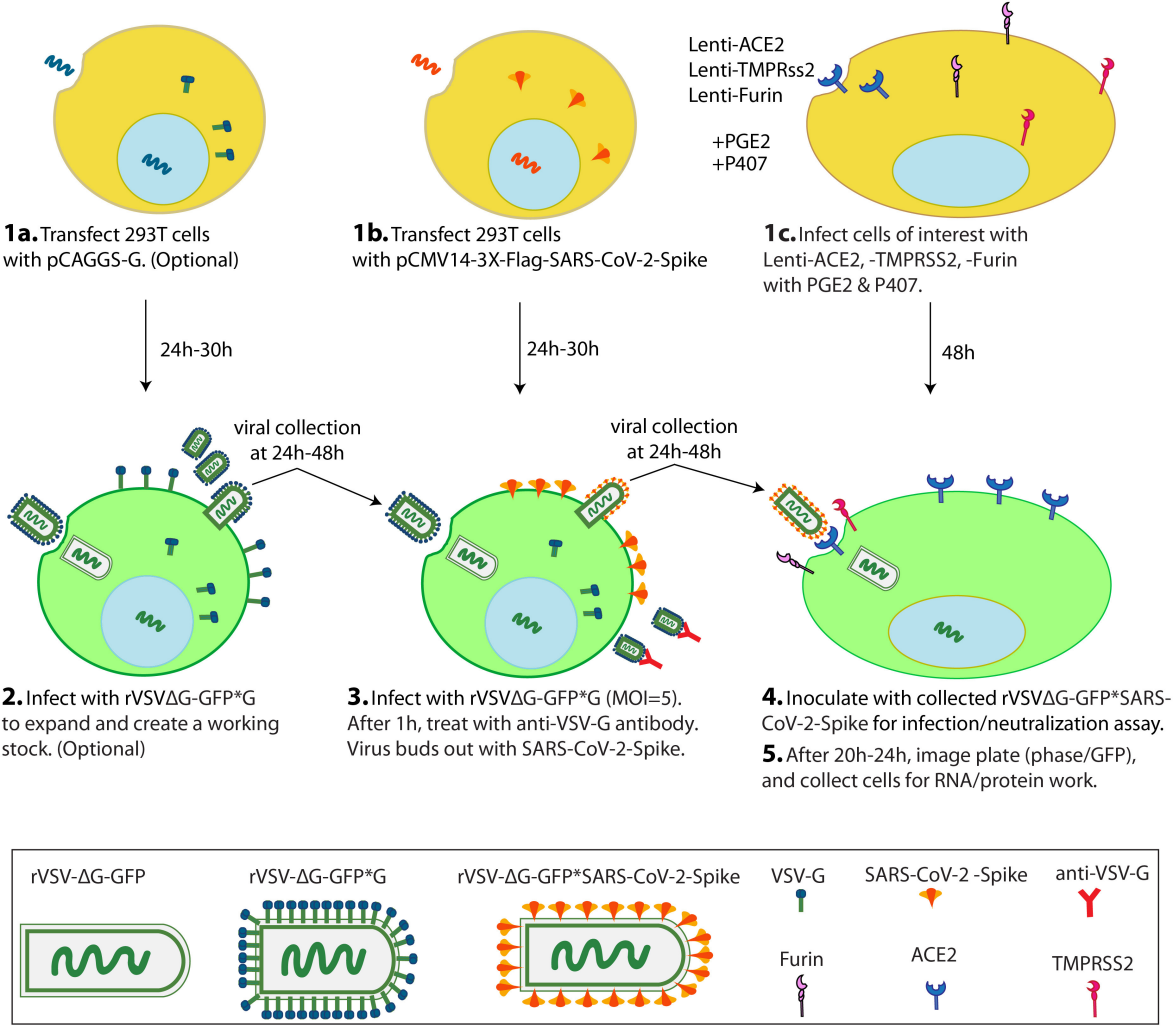
Scheme of VSV-ΔG*SARS-CoV-2 spike generation and its use in cells of interest in a 1-week protocol. On Day1, 2 transfections and 1 lentiviral infection are simultaneously performed. 293T cells are transfected with pCAGGS-G (1a), 293T cells are transfected with pCMV-3X-Flag-SARS-CoV-2 Spike (1b), and cells of interest (eg Vero-E6 cells) are infected with Lenti-ACE2, -TMPRSS2 and -Furin with PGE2 and P407 as adjuvants (1c). On Day 2, the 293T cells from 1a are inoculated with rVSVΔG-GFP*G to create an amplified working (2), and the 293T cells from 1b are inoculated with rVSVΔG-GFP*G from (2) or with a previously generated working stock of rVSVΔG-GFP*G to generate the pseudotyped rVSVΔG-GFP*SARS-CoV-2 virus (3). On Day 3 or 4, pseudotyped rVSVΔG-GFP*SARS-CoV-2 particles are collected and inoculated into the cells from 1c. On Day 5, scan the plate for GFP signal and finally collect cells for RNA/protein work (5). PGE2: Prostaglandin E_2_. P407: Polaxamer 407.

Note: BHK-21 cells can alternatively be used for pseudotyped viral constructs production (22).

This protocol is to generate VSV pseudoviruses bearing S protein (rVSV-GFPΔG*Spike) in 293T cell line with which to inoculate cells of interest for infection and neutralization assays (Figure 1). This protocol is based on the overexpression of a recombinant SARS-CoV-2 spike protein followed by pseudotyped VSV particle production by treating the spike protein expressing cells with rVSVΔG*G. In this protocol we used already produced rVSVΔG*G virus but these viral particles can be produced using the protocol of Whitt (22).

1. Plate 8–9 × 10^6^ 293T cells in a 100 mm tissue culture dish so that they are ~80% confluent on the day of transfection. The 293T cells are cultured in DMEM supplemented with 10% FBS and 1% P/S/G.

2. At the time of transfection, cells should look flat and be evenly distributed without clumps. Replace the growth medium with ~8 ml Opti-MEM™ or serum-free DMEM (without P/S/G or FBS) and incubate for ~20–30 min at 37°C while transfection mix is being prepared.
3. Preparation of the transfection mix:
  - Add 1.5 ml Opti-MEM to a 15 ml polypropylene tube, then add 16 μg pCMV14-3X-Flag-SARS-CoV-2 S, vortex gently, and incubate for 5 min at RT.
  - In another tube, dilute 48 μl Lipofectamine™ 2000 in 1.5 ml Opti-MEM™, vortex briefly, and incubate for 5 min at RT. Following the incubation, add the lipid mix to the DNA-OptiMEM™ mix, vortex gently, and incubate for 20 min at RT.
4. Remove the Opti-MEM™ or serum-free DMEM from the 293T cells and gently add the transfection mix directly to cells.
5. Rock the plate 4–5 times and bring the final volume of the media up to 4–5 ml by adding additional Opti-MEM™ or serum free DMEM media. Incubate the plates containing the transfection mix for 4–5 h at 37°C in a 5% CO_2_ incubator.
6. Following the 4–5 h incubation, remove the transfection mix and replace with complete growth medium (DMEM supplemented with 10% FBS). Incubate the cells at 37°C with 5% CO_2_.
7. Examine the cells beginning ~24 h post-transfection until the monolayer reaches confluency.
8. Once the transfected cells have reached confluency (~24–30 h post transfection), add the rVSVΔG*G virus [from Kerafast or produced in the laboratory according to Whitt (22)] at a multiplicity of infection (MOI) = 5, ~4–5 ×10^7^ IU (Infectious Units) in complete DMEM media to a final volume of 4 ml. The spike protein-transfected cells treated with the rVSVΔG*G virus are incubated at 37°C in 5% CO_2_.

Note: IU or Infectious Units is the titer of the virus. Because the pseudotypes undergo a single-round of infection, they don't produce plaques, but instead just infect a single cells, so we use the term infectious units rather than Plaque Forming Units (PFU). In addition, an MOI = 5 ensures that ~99.3% of the cells are infected based on a normal Poisson distribution. We found this to be optimal for production of VSV pseudotypes. Using a higher MOI can cause more rapid cell killing since a higher number of cells are receiving more than 1 infectious unit. A higher MOI also reduces the total amount of pseudotype virus produced ostensibly because the cells are undergoing apoptosis before maximal pseudotype virus production occurs.

9. After 1 h, the virus containing media is removed, the cells are washed carefully with PBS or serum-free DMEM, and the media replaced with 10 ml of complete DMEM media. Anti-VSV-G (Clone 8G5F11) (final concentration 1 μg/ml) is added to the media to neutralize residual rVSVΔG*G.
10. Incubate the cells for 24–48 h or until the cells start showing VSV-induced cytopathic effects (CPE) or >80–90% of the cells express GFP. VSV induces rapid cytopathic effects characterized by cell rounding and eventual cell death by apoptosis. [Critical Step].

Note: rVSVΔG*G viral particles can be purchased from Kerafast or produced using the protocol by Whitt (22). Briefly, 293T cells are plated in 100 mm dish and transduced with the VSV glycoprotein expression vector pCAGGS-G-Kan (16 μg) (Kerafast) using 80 μl of Lipofectamine™ 2000 using the approach outlined above (Steps 2–8). Approximately 24–30 h post transfection, the VSVG expressing cells are infected with rVSVΔG*G at MOI = 0.1. Twenty-four to Forty-eight hours post infection, the culture supernatant containing the expanded rVSVΔG*G virus is collected and the supernatant clarified to remove residual cells by either centrifuging the supernatant at ~500xg for 5 min. The clarified virus containing supernatant should be aliquoted in small volumes and stored at −80°C to avoid repeated freeze-thaw cycles. Generation of rVSVΔG*G working stock and rVSVΔG*SARS-CoV-2 Spike can be performed at the same day. We strongly recommend to produce rVSVΔG*G working stock, in at the least 2–100 mm dishes to produce sufficient virus for the experimental plan.

11. After 24–48 h post rVSVΔG*G infection, the supernatant containing the VSV pseudovirus carrying the spike protein of SARS-CoV-2 is harvested, clarified by centrifuging for 10 min at ~1,320 × g (slow deceleration mode). The virus should be used immediately or aliquoted into small batches and stored at −80°C.

Note: the virus containing supernatant should not be clarified by filtration.

12. Optional: Add 8 ml DMEM 10% plus 1 μg/ml of Anti-VSV-G for 24 h to obtain a second harvest.

**Figure 2:**
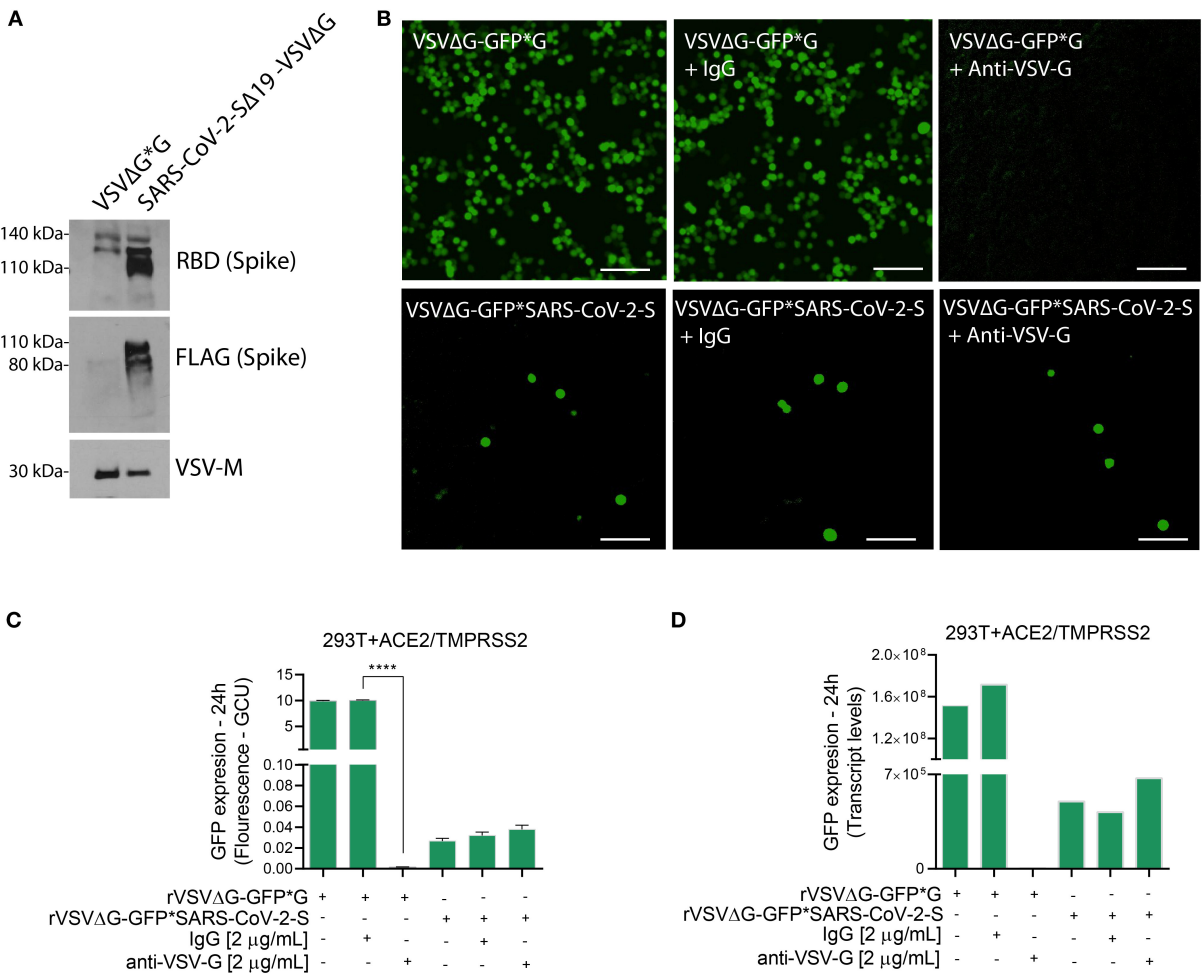
Characterization of VSVΔG*SARS-CoV-2 spike particles. **(A)** Immunoblotting for both rVSVΔG-GFP*G (control) and pseudotyped SARS-CoV-2 viruses were performed to verify the incorporation of FLAG-tagged Spike glycoprotein into the rVSVΔG. Shown are representative cellular images **(B)** of rVSVΔG-GFP*G and rVSVΔG-GFP*SARS-CoV-2 particle inoculation into 293T-ACE2+TMPRSS2 with and without Anti-VSV-G (2 μg/mL). After 24 h, the GFP signal from 25 images per well was quantified from IncuCyte scan, and normalized to cellular area **(C)**, and by qPCR from a single well and normalized to GAPDH **(D)**. Infection values are presented as % GCU to signal from corresponding control group, or as transcript levels. GCU: Green Calibrated Units. Scale bar: 100 μm. Data are mean ± SEM. ****p < 0.0001 using One-Way ANOVA with Tukey's *post-hoc* test. Each experiment was replicated at least three times.

Note: Use the supernatants of rVSVΔG*G and rVSVΔG*SARS-CoV-2 Spike for immunoblotting to confirm the spike incorporation into the rVSVΔG virus. Also, establish a neutralization assay using anti-VSV-G as part of proof of concept (Figure 2).

### Generation of Lentivirus for Over-expression of Human ACE2, TMPRSS2, and Furin in Cells of Interest

**Figure 3:**
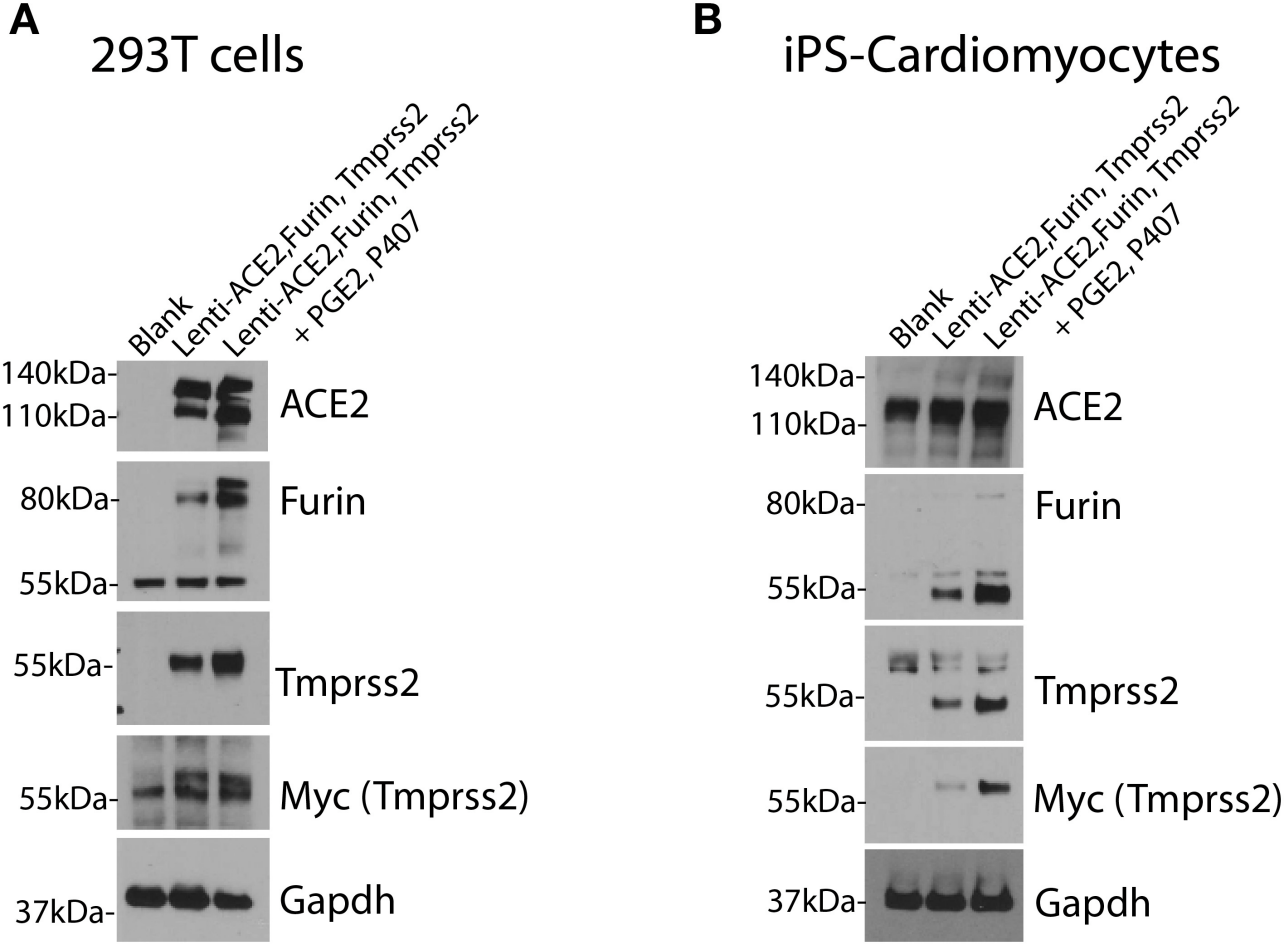
Over-expression of human ACE2, TMPRSS2 and Furin in cells of interest. 293T cells **(A)** and iPS-CMs **(B)** were infected simultaneously with lentivirus for human ACE2, Tmprss2, and Furin genes with and without the PGE2 and P407. Immunoblotting was performed after 48 h. Each experiment was replicated at least three times.

Note: Do not add antibiotics to the Packaging media.

For efficient infection of SARS-CoV-2 pseudovirus in cells of interest, the cells need to express the machinery needed for Spike protein recognition and priming. Therefore, cells of interest need to over-express human ACE2, TMPRSS2, and Furin as shown in Figure 3.

1. In 3 or 4 100 mm tissue culture dishes plate 8–9 × 10^6^ 293T cells in complete DMEM media (DMEM supplemented with 10% FBS and 1% P/S). The cells should be ~80–90% confluent at the time of transfection.
2. On the day of transfection, replace the complete media with 8 ml of OptiMEM and prepare the plasmid mixture (per plate) as following:
  - Lentivirus construct−6 μg
  - psPAX2 (lentiviral packaging plasmid)−4 μg
  - pMD2.GVSV-G (VSV glycoprotein expression plasmid)−2 μg.
    Add the required volume of each plasmid to 1.5 ml of OptiMEM™ media. Simultaneously, add 36 μl of Lipofectamine™ 2000 to 1.5 ml of OptiMEM™ medium in a separate tube. Mix with gentle pipetting. Incubate both the DNA containing media and the Lipofectamine™ 2000 containing media at room temperature. After 5 min, add the DNA containing solution with the Lipofectamine™ 2000 containing solution and mix gently. Incubate the transfection mix for 20 min at room temperature.
3. Aspirate the media from the 293T cells and gently add the transfection mix. Rock the plates back and forth 4–5x and add 2 ml more of Opti-MEM™ to bring the volume up to 5 ml.
4. Incubate the cells at 37°C in 5% CO_2_ for 4–5 h, aspirate off the transfection media, and replace with 10 ml of DMEM supplemented with 5% FBS and 0.2% Sodium Pyruvate (Packaging media).

5. Incubate the plates overnight at 37°C with 5% CO_2_. The following day (24 h after transfection), collect the medium, store at 4°C, and add 10 ml of Packaging media (see above) to the cells.
6. Monitor mCherry expression following the transfections (~24–48 h). For optimal viral titers, >80–90% of the cells should be mCherry-positive. If mCherry+ cells are <50%, transfections, the transfection should be performed again.
7. Collect the supernatant at 48 h in 50 ml conical tubes (Second harvest), save at 4°C, and replace with 10 ml DMEM (packaging media).
8. After 72 h post transfection, collect the supernatant from the cells (Third harvest) and pool the supernatants from all three harvests and clarify the supernatant by centrifugation (1,000xg for 10 min), carefully collect the supernatant, and passage through a 0.45 μm filters.
9. The viral particles can be concentrated by overlaying the clarified supernatant on a 10% sucrose-containing buffer [50 mM Tris-HCl, pH 7.4, 100 mM NaCl, 0.5 mM ethylene diamine tetra acetic acid (EDTA)] (29) at a 4:1 v/v ratio and centrifuge at 60,000xg for 2.5 h at 4°C (slow deceleration mode).

Note: Alternative methods can be used to concentrate the virus in the absence of an ultracentrifuge, such as using the LentiX™ concentrator solution (Takara Bio).

10. After centrifugation, the supernatant is removed in a biosafety cabinet and the tube is placed inverted on a clean tissue paper for 3 min. Add 200 μl of PBS as final volume and thoroughly resuspend the pellet. Aliquot the resuspended virus into fresh tubes (20 μl/tube) and store at −80°C until needed.

Note: Avoid freeze–thaw cycles because it reduces considerably the lentiviral titer. Use ice to thaw the small aliquots.

11. The lentivirus can be titered by applying serial dilutions of the concentrated virus to 293T cells. Plate 293T cells in a 24-well-plate to obtain ~80% confluence. Make 10-fold serial dilutions from 2 × 10^−2^ (1:50) of the virus stock up to 2 × 10^−7^ (1:5,000,000) by adding the virus into DMEM supplemented with 2% FBS. Add 490 μl of DMEM + 2% FBS in the first tube and 450 μl in the others five tubes. Add 10 μl of concentrated lentivirus in the first tube, mix by pipetting up and down several times and add 50 μl of first mixture to the second tube. Continue to dilute the virus using the remaining tubes containing DMEM + 2% FBS. Replace media from the 24-well plates with each tube containing the diluted virus. After 24 h, replace the virus containing media with DMEM + 10% FBS. Monitor for the expression of mCherry for 48–72 h post infection. Count the number of mCherry+ cells in the wells treated with the different amounts of virus using a Fluorescent microscope. Using the well with between 5 and 15 mCherry+ cells will be used to calculate TU (TU/mL: [#mCherry+ cells] × [Dilution factor] × 100).

Note: Use this method for mCherry-containing Lentivirus like the Lenti-ACE2-mCherry and Lenti-TMPRSS2-mCherry used here. For lentiviral constructs that lack a fluorescent protein (e.g., Lenti-Furin), a commercial lentiviral titering kit [e.g., Lenti-X™ qRT-PCR Titration Kit (Takara Bio)] can be used.

12. Generation of stable cell lines. In a 24-well plate, seed 293T cells or any desired cell line to obtain a confluence of ~80%. At the time of infection, add Poloxamer 407 (100 μg/mL) and PGE2 (10 μM), then add Lenti-ACE2, Lenti-TMPRSS2 and Lenti-Furin at MOI = 2–3 or at 1 × 10^6^ TU/mL. After 48–72 h expand the cells or use for inoculation with SARS-CoV-2 pseudovirus.

The present protocol uses 2 adjuvants to improve lentiviral transduction. Poloxamer 407 (P407) is a hydrophilic non-ionic surfactant, which together with other poloxamers are commonly used to improve drug delivery and as an adjuvant in several cells lines since they can act by membrane fluidization, increasing lipid exchange and reducing membrane viscosity (30, 31). PGE2 acts by enhancing intracellular cAMP leading to an improvement in viral transduction. The combination of these two molecules (P407 and PGE2) has been previously shown improve lentiviral transduction in CD34+ cells with high-efficiency and without significant cytotoxicity (32).

Notes:
*Since the Lenti-ACE2, -TMPRSS2, and –Furin vectors all contain a puromycin resistance gene, stable cell lines can be generated by treating the transduced cells with 3 μg/ml of puromycin for 48 h.
*Poloxamer 407: Prepare in PBS 1x (5 mg/ml). Reagent should be open in the biosafety cabinet and filtered (0.22 μm).
*PGE2: Prepare aliquots of 10 mM PGE2 in DMSO (Dimethyl sulfoxide) from a 1 mg PGE2 stock. Since PGE2 is prepared using methyl acetate, the methyl acetate needs to be evaporated prior to resuspending in the DMSO. This can be accomplished by gently pipetting air into the vial until completely evaporated. Resuspend the PGE2 in 263 μl of DMSO and pipette up and down to mix. Aliquot the resuspended PGE2 into clean, sterile microcentrifuge tubes and store at −80°C until needed. Prepare working stock solutions at 1 mM PGE2 in PBS.

### SARS-CoV-2 Pseudovirus Infection for Viral Entry and Neutralization Assays

1. In a 24-well plate, seed 293T, Vero-E6 cells or any desired cell line which have been engineered to overexpress ACE2/TMPRSS2, ACE2/Furin, or ACE2/TMPRSS2/Furin (see above) to obtain a confluence of 80%.

Note: For titration purposes of SARS-CoV-2 pseudovirus, seed 293T-ACE2/TMPRSS2 or Vero-E6 ACE2/Furin overexpression cells in a 24-well-plate, make 3-fold serial dilutions up to 1:243 using DMEM/MEM 2% FBS as diluent. After 24 h, count the number of GFP+ cells in the last wells to calculate the TU/mL. Usually, 100–200 μl of supernatant containing the VSV-SARS-CoV-2 pseudovirus are enough to perform the experiments.

2. Viral entry assay: Replace the medium with 200–300 μl cell culture medium (DMEM or MEM) supplemented with 2% FBS and 1% P/S/G and perform the treatments with known SARS-CoV-2 blockers (as positive controls) such as Camostat mesylate, a TMPRSS2 inhibitor (20–50 μM)(3), Hydroxychloroquine (20–50 μM) (33), anti-ACE2 (10–20 μg/mL) or other treatments of interest for 20 min.

Note: This method of inoculation yields to moderate infection levels, an inherent characteristic of the SARS-CoV-2 when used to pseudotype either VSV or HIV, as shown by others (3, 34).

**Figure 4:**
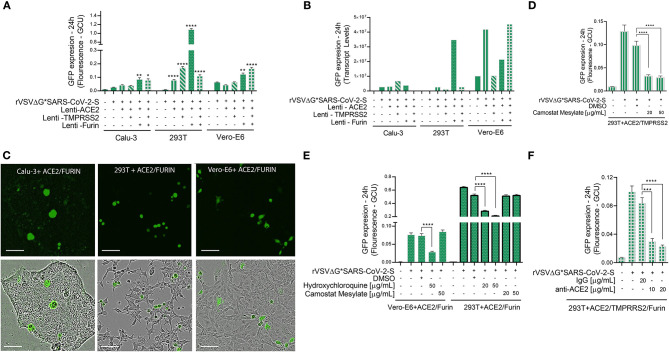
Viral entry assay in 3 cell lines over-expressing ACE2, TMPRSS2 and/or Furin. Calu-3, 293T cells, and Vero-E6 cells—all over-expressing ACE2, Tmprss2, and/or Furin were inoculated with VSV-ΔG*SARS-CoV-2 spike particles. After 24 h, the GFP signal from 25 images per well was quantified from IncuCyte scan, and normalized to cellular area **(A)**, and then by qPCR from a single well **(B)**. Shown are representative cellular images from IncuCyte scan **(C)**. Infection was quantified from 25 images per well after treatment with anti- ACE2, Hydroxychloroquine or Camostat Mesylate, 20 min before inoculation in 293T and Vero-E6 cells with ACE2, TMPRSS2 and/or Furin over-expression **(D–F)**. Infection was also quantified after treatment with anti-ACE2 antibody or IgG control in 293T cells with ACE2, TMPRSS2, and Furin over-expression. **(E)**. Values are presented as Green Calibrated Units (GCU) normalized to cell area, or mRNA transcripts normalized to GAPDH, respectively. Scale bar: 100 μm. Data are mean±SEM. *p < 0.05; **p < 0.01; ***p < 0.001; ****p < 0.0001 - using One-Way ANOVA with Tukey's *post-hoc* test. Each experiment was replicated at least three times.

**Figure 5:**
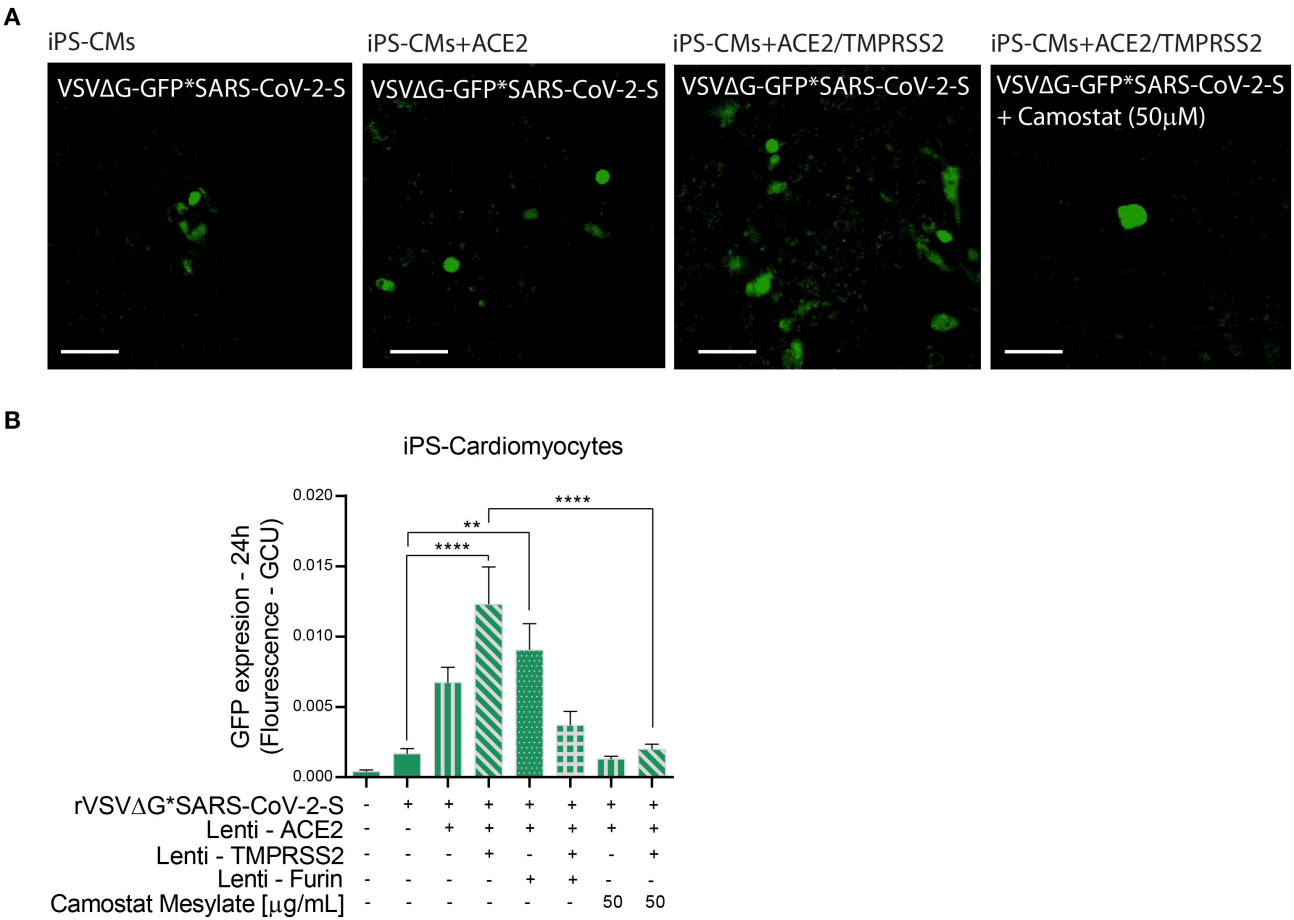
Viral entry assay in beating iPS-CMs over-expressing ACE2, TMPRSS2 and/or Furin. Human iPS-CMs over-expressing ACE2, Tmprss2, and/or Furin were inoculated with VSVΔG*SARS-CoV-2 spike particles. Shown are representative cellular images from IncuCyte scan **(A)**. After 24 h, the GFP signal from 25 images per well was quantified from IncuCyte scan, and normalized to cellular area. Camostat Mesylate treatment was performed 20 min before inoculation. Values are presented as Green Calibrated Units (GCU) normalized to cell area **(B)**. Scale bar: 100 μm. Data are mean ± SEM. **p < 0.01; ****p < 0.0001 - using One-Way ANOVA with Tukey's *post-hoc* test. Each experiment was replicated at least three times.

**Figure 6:**
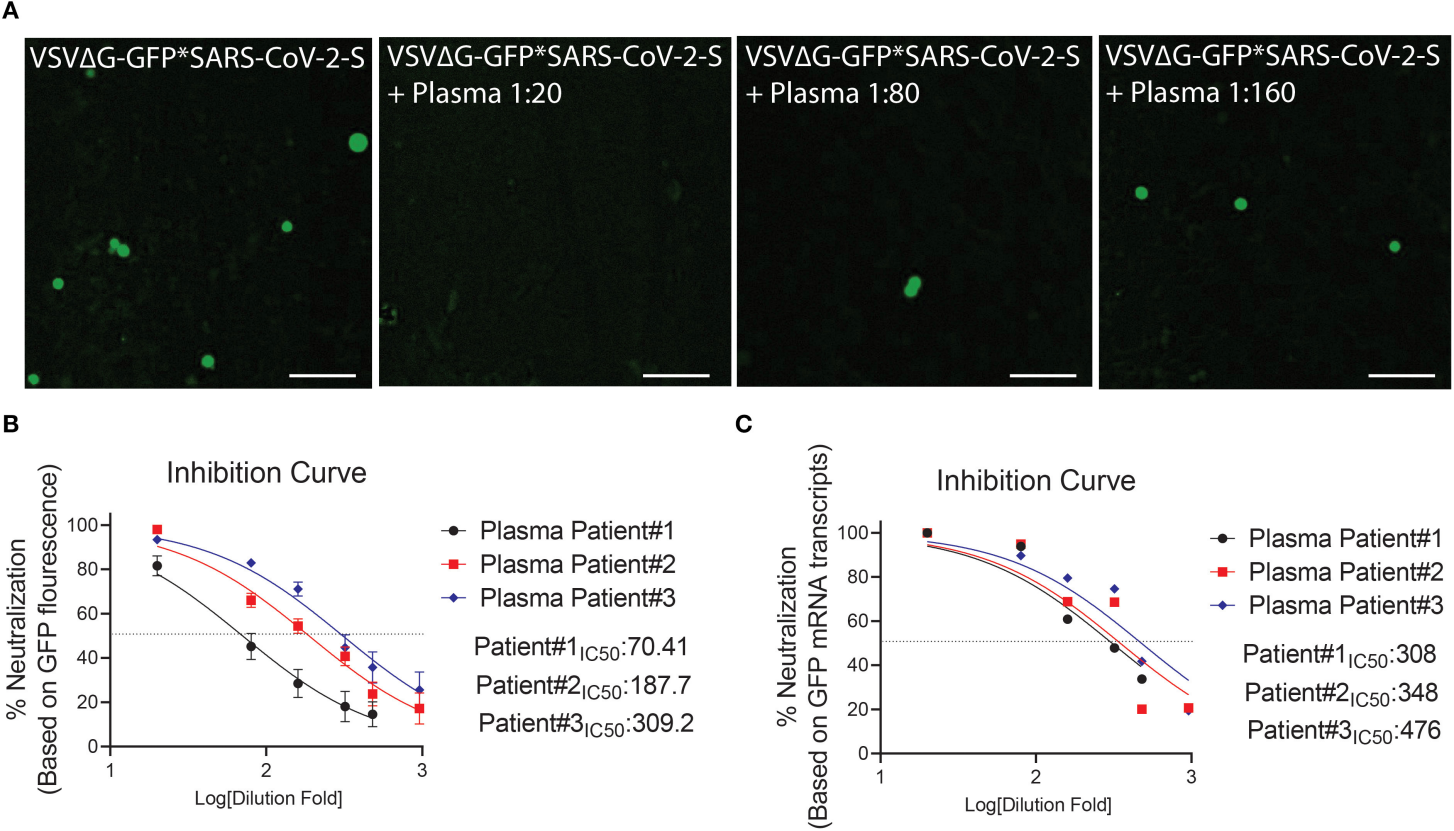
Neutralization assay using plasma from COVID-19 surviving patients. **(A)** Shown are representative cellular images from IncuCyte scan. **(B,C)** Inhibition curve was performed with convalescent plasma at serial dilutions starting from 1:20 to 1:960. After 24 h, the GFP signal from 25 images per well was quantified from IncuCyte scan, and normalized to cellular area **(B)**, and then from a single well by mRNA transcripts **(C)**. Inhibition curves are presented in log-transformed dilution with IC_50_ values for each patient. IC_50_: Half maximal inhibitory concentration. Each experiment was replicated at least three times.

3. Inoculate with the SARS-CoV-2 spike protein pseudotyped VSVΔG by adding 100–200 μl of complete media to bring the final volume to 400 μl.
4. Twenty to twenty four hours post infection, scan the plates using the Incucyte ZOOM imaging system (Sartorius) and analyze the images (Figures 4, 5). See Live Cell Image Acquisition and Analysis section.
5. Neutralization assay using plasma from recovered COVID-19 patients. Serial dilutions were made of convalescent plasma obtained from individuals who had recovered from SARS-CoV-2 infection. Two-fold dilutions were made in DMEM supplemented with 2% FBS (range of 1:20 through 1:960 dilutions). This is achieved by adding 360 μl of DMEM 2% FBS in the first tube and 200 μl in the others five tubes. Forty microliter of plasma from recovered COVID-19 patients was added to the first tube, mixed, and 200 μl of first mixture was added to 200 μl of DMEM + 2% FBS for the remaining tubes to get the full dilution range. The media is aspirated from the cells and replaced with 200 μl of the diluted plasma/DMEM + 2% FBS mixture. Incubate for 20 min at 37°C in 5% CO_2_.
6. Inoculate with 200 μl of the SARS-CoV-2 spike protein pseudotyped VSVΔG virus to each well and incubate at 37°C with 5% CO_2_.
7. Twenty to twenty four hours post infection, scan the plates using the IncuCyte ZOOM imaging system, and analyze the images (Figure 6). See Live Cell Image Acquisition and Analysis section.

### Live Cell Image Acquisition and Analysis

1. Twenty to twenty four hours post infection, scan the plates with a 10X objective using the IncuCyte ZOOM imaging system.
2. Open the Incucyte^TM^ ZOOM software and set up the system to obtain 25 images per well using the green and phase channels.
3. When the scanning is complete, create an image set to quantify all parameters of phase-contrast area as well as fluorescent measures. Divide the Total Green Object Integrated Intensity [Green Calibrated Units (GCU) × μm^2^/image] values of each image by its corresponding Total Phase Area (μm^2^/image) to obtain the normalized GFP expression (GCU) values per image. Detailed protocol for IncuCyte operation and analysis was described by our group (35).

### Collection of Cells for RNA and Protein Work

1. After scanning, the cells can be washed in cold PBS, scraped, and collected in 80–100 μl RIPA Lysis buffer and Extraction Buffer or Cell Disruption Buffer from the Mirvana Kit, and used for RNA extraction for qPCR or for immunoblotting. It is important to collect the cells immediately after the IncuCyte scan for accurate comparison between GFP expression by immunoflourescence vs. qPCR. In case of delays between the scan of the plate and the qPCR, there will be some differences in the signal from both methods.
2. For qPCR, extract RNA from cell lysates using Mirvana Paris Kit or an alternative kit, and perform reverse transcription and cDNA amplification using a cDNA kit. Quantify gene transcripts by qPCR using Taqman assays for GFP and GAPDH and a “Fast” qPCR mastermix on an ABI 7900HT thermocycler. For each group, plot fluorescence against the number of cycles on a logarithmic scale and normalize data normalized to the endogenous GAPDH control. The normalized Cycle Thresholds (delta CTs) can be converted to absolute transcripts using an equation where a CT of 40 corresponds to 1 transcript.

## Data Availability Statement

The raw data supporting the conclusions of this article will be made available by the authors, without undue reservation.

## Ethics Statement

The studies involving human participants were reviewed and approved by University of Miami IRB. The patients/participants provided their written informed consent to participate in this study.

## Author Contributions

JC and GL performed the experiments. JC and LS analyzed the data and prepared the figures. JC, GL, and LS interpreted the results of experiments. JC drafted the manuscript. GL, DD, AS, JH, MW, SP, DJ, and LS edited and revised the manuscript. JC, GL, DD, AS, JH, MW, SP, DJ, and LS approved the final version of the manuscript. All authors contributed to the article and approved the submitted version.

## Conflict of Interest

The authors declare that the research was conducted in the absence of any commercial or financial relationships that could be construed as a potential conflict of interest.
